# CD4^+^ T-cell responses to foot-and-mouth disease virus in vaccinated cattle

**DOI:** 10.1099/vir.0.045732-0

**Published:** 2013-01

**Authors:** B. Veronica Carr, Eric A. Lefevre, Miriam A. Windsor, Cristina Inghese, Simon Gubbins, Helen Prentice, Nicholas D. Juleff, Bryan Charleston

**Affiliations:** 1 Pirbright Institute, Compton Laboratory, Compton, Newbury, Berkshire, RG20 7NN, UK; 2 Pirbright Institute, Pirbright Laboratory, Ash Road, Woking, Surrey, GU24 0NF, UK

## Abstract

We have performed a series of studies to investigate the role of CD4^+^ T-cells in the immune response to foot-and-mouth disease virus (FMDV) post-vaccination. Virus neutralizing antibody titres (VNT) in cattle vaccinated with killed FMD commercial vaccine were significantly reduced and class switching delayed as a consequence of rigorous *in vivo* CD4^+^ T-cell depletion. Further studies were performed to examine whether the magnitude of T-cell proliferative responses correlated with the antibody responses. FMD vaccination was found to induce T-cell proliferative responses, with CD4^+^ T-cells responding specifically to the FMDV antigen. In addition, gamma interferon (IFN-γ) was detected in the supernatant of FMDV antigen-stimulated PBMC and purified CD4^+^ T-cells from vaccinated cattle. Similarly, intracellular IFN-γ could be detected specifically in purified CD4^+^ T-cells after restimulation. It was not possible to correlate *in vitro* proliferative responses or IFN-γ production of PBMC with VNT, probably as a consequence of the induction of T-independent and T-dependent antibody responses and antigen non-specific T-cell responses. However, our studies demonstrate the importance of stimulating CD4^+^ T-cell responses for the induction of optimum antibody responses to FMD-killed vaccines.

## Introduction

The contribution of T-cells in the host response to foot-and-mouth disease virus (FMDV) vaccination is not well understood. FMDV, a picornavirus, is the cause of an acute, highly contagious and economically important disease of cloven-hoofed animals. Control measures include slaughter, restrictions on the movement of livestock and vaccination of susceptible animals. Disadvantages with current vaccines include the insufficient block of virus shedding, failure to prevent virus carriers, short duration of immunity and difficulty in distinguishing vaccinated from infected animals (Paton *et al.*, 2009). Current inactivated FMD vaccines that have been developed empirically generate short-term, serotype-specific protection, mainly through neutralizing antibodies and protection is generally correlated with high levels of neutralizing antibodies (McCullough *et al.*, 1992). With increased interest in the production of new improved vaccines, growing attention has been applied to the issue of specific lymphocyte memory, its generation, and the properties of the cells involved. It is becoming increasingly clear that the next generation of vaccines to be designed will require a more complete understanding of the mechanisms and processes that give rise to immunological memory.

CD4^+^ T-cells play an important role in establishing and maximizing the capabilities of a number of arms of the immune system, such as helping B-cells produce high affinity antibodies against antigens, inducing macrophages to develop enhanced microbicidal activity and recruiting neutrophils, eosinophils and basophils to sites of infection and inflammation (Zhu & Paul, 2008).

Without participation by these CD4^+^ T-cell subsets, B-cells cannot undergo isotype switching to generate high-affinity antibodies, the microbicidal activity of macrophages is reduced, the efficiency of CD8^+^ T-cell responses and CD8^+^ T-cell memory are compromised, and downregulation of effector responses is impaired (Stockinger *et al.*, 2006). Therefore, CD4^+^ T-cells are likely to fulfil an important facilitator role in the maintenance and control of protective immune responses against FMDV.

The importance of neutralizing antibodies in FMDV infection has been reviewed extensively (Becker, 1994; Brown, 1995; Collen, 1994). Since the protection afforded by most vaccines at present depends to a large extent on humoral immunity, it is appropriate that much attention has been focused on the generation of B-cell memory and long-lived plasma cells driven by antigen-specific CD4^+^ helper T-cells. Some investigators have shown that protection correlates to neutralizing antibody titre levels, whilst others have reported that some vaccinated animals with little or no detectable neutralizing antibody are resistant to challenge with live virus (DiMarchi *et al.*, 1986). A number of publications describe the induction of specific CD4^+^ T-cell responses against FMDV, for example: CD4 epitopes within both proteins P1A and P1D have been identified in cattle (Gerner *et al.*, 2007) and FMDV-specific MHC class II-restricted responses have been described in cattle (Glass *et al.*, 1991; Guzman *et al.*, 2008) and pigs (Gerner *et al.*, 2006). However, the dependence of protective antibody responses on the induction of specific CD4^+^ T-cell responses has not been demonstrated.

In this paper, we examine the role of T-cell subsets during FMD vaccination. First, we investigated whether specific CD4^+^ T-cell responses are required to support the production of virus neutralizing antibodies. We then studied in detail the specificity of some of the assays currently used to measure T-cell responses to FMDV. Finally, we investigated whether there was a correlation between specific CD4^+^ T-cells responses and the magnitude of antibody responses.

## Results

### *In vivo* depletion of CD4^+^ T-cells and antibody responses post-FMD vaccination

**Fig. 1.**
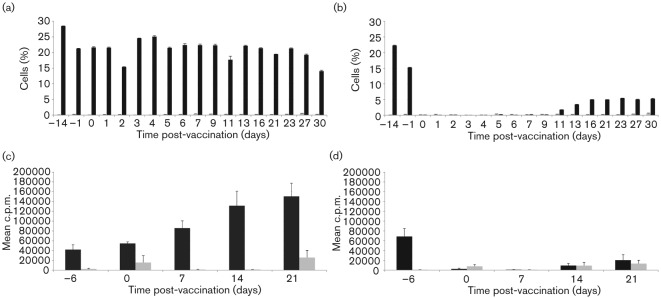
Intravenous administration of CD4 mAbs results in a transient depletion of circulating CD4^+^ T-cells in peripheral blood and loss of proliferative response to BHV-1 antigen. Two groups of three calves were vaccinated with commercial BHV-1 vaccine, and subsequently vaccinated with A22 Iraq FMD commercial vaccine (2–3 weeks later). Each animal received intravenous injections of either an isotype-matched control TRT 3 (a and c) or anti-CD4 (b and d) mAb for a period of 4 days, starting the day before FMD vaccination. At various time points prior to and following FMD vaccination, the percentage of CD4^+^ T-cells in PBMC was determined by flow cytometry after staining the cells with either an anti-CD4 (black bars) or TRT1 isotype control (grey bars) mAb (a and b) and the proliferation of PBMC to heat-inactivated BHV-1(1/100, black bars) or EBTr cell lysate (control antigen, 1/100, grey bars) was assessed by [methyl-^3^H] thymidine incorporation (c and d). Results are expressed as the mean of determinations from individual calves in each group±sem, *n* = 3.

**Fig. 2.**
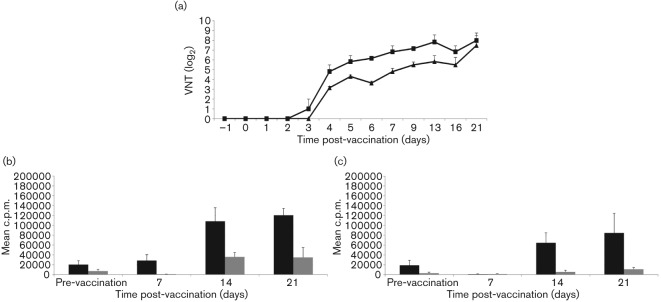
Effect of the CD4^+^ T-cell depletion on VNT and proliferation to FMDV vaccine antigen. Two groups of three calves were treated as described in Fig. 1. (a) VNTs are shown for anti-CD4 mAb (triangles) and TRT3 mAb-(squares) treated calves at time points pre- and post-vaccination. Results are expressed as the mean of determinations (log_2_ titres) from individual calves in each group±sem. (b) Total PBMC proliferative responses are shown for TRT3 mAb-treated calves to A22 FMDV vaccine antigen (0.1 µg ml^−1^, black bars) or BHK-21 cell lysate (0.1 µg ml^−1^, grey bars) at time points pre- and post-vaccination, as assessed by [methyl-^3^H] thymidine incorporation. Results are expressed as the mean of determinations from individual calves in each group±sem. (c) Similar to (b), but showing the antigen-specific proliferation of PBMC from CD4 mAb-treated calves. Results are expressed as the mean of determinations from individual calves in each group±sem.

The administration of cc8, but not of the isotype control TRT3, mAb resulted in the efficient reduction of circulating CD4^+^ T-cells to undetectable levels when measured by flow cytometry in blood (Fig. 1a and b) and lymph nodes (data not shown). This CD4-depletion was transient as CD4^+^ T-cells began to be detected again in peripheral blood by day 11 (Fig. 1b). For the duration of the experiment, the non-depleted animals retained functional capacity to respond to the T-dependent antigen, BHV-1 (Fig. 1c), whilst functional depletion was demonstrated by the abrogation of pre-established T-cell responses to BHV-1 vaccination in the CD4-depleted animals (Fig. 1d). FMDV-specific virus neutralization titres (VNTs) were significantly (*P*<0.05) higher in non-depleted compared with CD4-depleted cattle on days 5, 6, 7 and 9 (VNT, Fig. 2a). Similarly, FMDV-specific IgG1, IgG2, IgM antibodies and G-H loop-specific antibodies were significantly (*P*<0.05) higher in non-depleted compared with CD4-depleted cattle on days 9 and 13, days 7 and 9, days 6, 7 and 9 and days 13 and 16, respectively (Table S1, available in JGV Online). These differences at individual time points broadly reflect the results of the parameter estimation, with significant (*P* = 0.05) differences between CD4-depleted and non-depleted cattle in the timing of the maximum rate of increase (δ) of antibody production (Table 1). Although IgM antibodies can be produced in the absence of T-cell help, it is not unusual to observe higher IgM titres when T-cell help is available. Indeed, similar results have been described by Haberthur *et al.* with a rhesus macaques model where CD4^+^ depletion followed by a challenge with simian varicella virus resulted in reduced IgM titres (Haberthur *et al.*, 2011). The maximum rate of increase (δ) in IgG1, IgG2, IgM and G-H loop antibody levels occurred significantly (*P* = 0.05) earlier for the non-depleted compared with the CD4-depleted cattle (Table 1). At day 7 post-FMD vaccination, a specific proliferative response to FMDV antigen was detected in the non-depleted, but not in the CD4-depleted animals. However, once CD4^+^ T-cells were detectable in the circulation of depleted animals, the magnitudes of proliferative responses to FMDV antigen between the depleted and non-depleted animals were comparable on days 14 and 21 post-vaccination (Fig. 2b and c). T-cell proliferative responses and VNTs were not significantly correlated (Spearman partial correlation = 0.34, *P* = 0.06), indicating a component of this antibody response is T-cell independent.

**Table 1.** Estimates for the timing of maximum rate of increase (δ) in FMDV-specific VNT, IgG1, IgG2, IgM and G-H loop peptide antibody levels for individual cattle Results are expressed as the time (in days) at which the maximum rate of antibody level increase occurred post-FMD vaccination.

	Non-depleted group			CD4^+^ T-cell-depleted group		
	FMD 47	FMD 49	FMD 50	FMD48	FMD 51	FMD 52
VNT	3.8	2.9	4.0	4.4	4.5	3.6
IgG1	7.9	6.6	5.8	8.2	8.1	15.3
IgG2	4.9	6.1	4.0	6.9	8.4	15.1
IgM	4.0	3.6	3.7	5.0	8.6	16.0
G-H loop peptide	12.7	6.7	8.1	19.1	13.0	–*

* No increase in G-H loop peptide ELISA levels were observed for this animal.

### Neutralizing antibodies and proliferative responses post-FMD vaccination

We determined the kinetics of the neutralizing antibody response following FMD O1 Manisa vaccination and boost-vaccination in 10 calves (FMD 1–FMD 10). FMDV-specific VNTs were not detected in animals before vaccination and were first detected 7 days post-vaccination in all animals (Table 2). Eight out of ten animals received a booster immunization at either weeks 6 or 11, resulting in a marked increase in VNTs by week 14. Subsequently, antibody titres were still detectable by week 30 post-primary immunization in all but four animals (FMD 1, 2, 3 and 5; data not shown). Cellular recognition of FMDV vaccine antigen was evaluated at various time points post-vaccination by assessing the proliferative cell-mediated responses of cultured total PBMC or purified CD4^+^ T-cells. A specific proliferative response could be detected to the FMDV vaccine antigen as early as day 7 in most animals (Table 2). This response was maintained throughout the course of the experiment (>12 months). The proliferative response (c.p.m.) and the magnitude of the antibody response (VNTs) at most of the time points examined were not significantly correlated (Spearman partial correlation = 0.14, *P* = 0.30). It is worth noting that throughout this series of experiments, the magnitude of responses varied greatly between individual animals. Evidence that this proliferative response was antigen-specific was demonstrated by titration of the response with decreasing antigen concentration *in vitro* and lack of response of PBMC to BHK-21 cell lysate (data not shown), which excludes the possibility that the response detected was against irrelevant cellular antigens present in the FMDV vaccine antigen preparations.

**Table 2.** Summary of vaccination regimes, total PBMC proliferation responses and maturation of the VNT antibody titres post-FMD vaccination

Animal no.	Vaccine	Boost* (weeks)	Day 0		Day 7		Day 14		Day 21		Day 28		Week 14	
			c.p.m. to FMD**†** (±sd)**‡**	VNT titre**§**	c.p.m. to FMD	VNT titre	c.p.m. to FMD	VNT titre	c.p.m. to FMD	VNT titre	c.p.m. to FMD	VNT titre	c.p.m. to FMD	VNT titre
FMD 1	O1 Manisa	6	12 500 (19 672)	Neg¶	9 266 (8 194)	45	6 134 (4 963)	64	1 932 (1 243)	22	48 748 (32 850)	64	60 470 (34 488)	178
FMD 2	O1 Manisa	6	1 432 (855)	Neg	15 478 (1 708)	45	5 736 (195)	64	45 881 (30 371)	90	142 719 (49 987)	128	242 269 (33 619)	708
FMD 3	O1 Manisa	nb||	1 817 (2 105)	Neg	420 551 (52 363)	90	91 252 (69 670)	128	42 937 (17 709)	256	21 877 (11 071)	90	142 370 (13 501)	90
FMD 4	O1 Manisa	6	2 338 (3 245)	Neg	357 162 (56 790)	64	346 342 (32 520)	128	416 495 (16 182)	256	308 855 (28 391)	178	502 141 (11 086)	1413
FMD 5	O1 Manisa	nb	1 103 (260)	Neg	130 436 (89 257)	64	333 068 (114 336)	128	216 857 (63 094)	90	346 991 (65 830)	90	162 680 (32 964)	45
FMD 6	O1 Manisa	11	81 671 (52 319)	Neg	165 255 (12 835)	90	50 369 (26 136)	90	113 014 (28 660)	178	159 564 (7 549)	256	26 178 (21 699)	1024
FMD 7	O1 Manisa	11	10 393 (10 232)	Neg	336 162 (13 014)	128	252 146 (32 180)	90	373 946 (48 571)	128	319 170 (56 258)	355	97 814 (35 847)	1413
FMD 8	O1 Manisa	11	14 742 (12 366)	Neg	277 555 (31 612)	64	234 206 (15 265)	128	173 348 (11 601)	178	243 187 (11 634)	512	82 680 (38 298)	1413
FMD 9	O1 Manisa	11	11 656 (16 688)	Neg	179 178 (11 081)	90	119 878 (58 750)	128	186 864 (43 490)	128	24 841 (16 140)	128	164 185 (17 244)	1413
FMD 10	O1 Manisa	11	32 886 (54 912)	Neg	157 599 (20 249)	90	72 538 (106 423)	128	145 232 (22 443)	128	169 403 (27 988)	355	68 447 (12 700)	1413

* Time post-vaccination at which animals received a boost vaccination with O1 Manisa commercial vaccine.

† Mean c.p.m. value of triplicate determinations to FMDV O1 Manisa vaccine antigen (0.1 µg ml^−1^).

‡ sd of the mean of triplicate determinations.

§ The final dilution of serum present in the serum/virus mixture that gives ≥50 % neutralization of virus growth, expressed as the mean of duplicate determinations.

||nb, Not boosted.

¶ Negative.

### Measurement of T-cell subset proliferation post-FMD vaccination

**Fig. 3.**
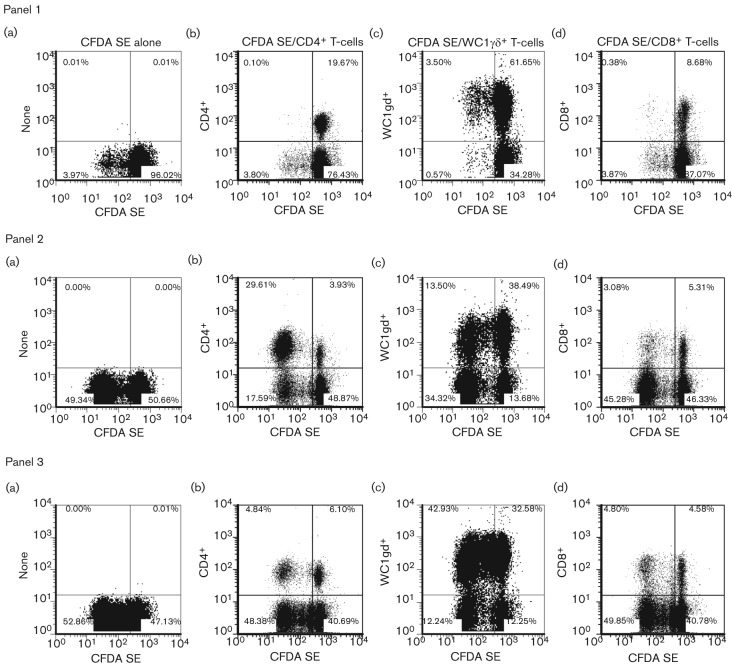
Proliferation of T-cell subsets monitored by CFDA SE labelling in response to *in vitro* restimulation with FMDV vaccine antigen and p252. PBMC from cattle previously vaccinated with O1 Manisa FMD commercial vaccine were labelled with CFDA SE prior to their culture *in vitro* for 6 days in the presence of medium alone (panel 1), inactivated O1 Manisa FMDV vaccine antigen (panel 2) or FMDV p252 (panel 3). At the end of the culture period, the cells were stained for expression of cell surface differentiation antigens without (a) or with APC-conjugated cc8 (CD4^+^ T-cells, b), cc15 (WC1^+^ γδ T-cells, c) and cc63 (CD8^+^ T-cells, d) mAbs, and analysed by flow cytometry. The percentages of cells in each quadrant are illustrated. One representative dataset (animal FMD 4) from three independent experiments using two animals is shown.

**Fig. 4.**
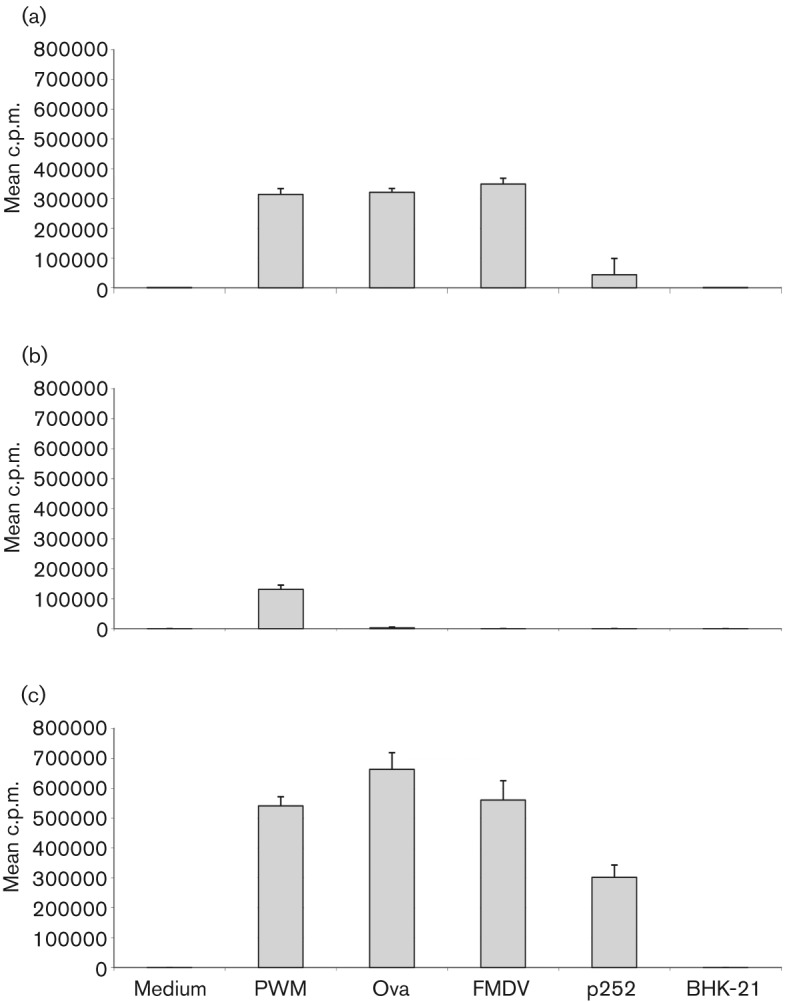
Proliferation post-vaccination monitored by thymidine incorporation in response to *in vitro* restimulation with FMDV vaccine antigen and p252. Calves were immunized with Ova in incomplete Freund’s adjuvant, and subsequently vaccinated with O1 Manisa FMD commercial vaccine (6 weeks later). Total PBMC (a), PBMC depleted of CD4^+^ T-cells (b) and purified CD4^+^ T-cells+APCs (c) were cultured *in vitro* in the presence of medium alone or optimal concentrations of PWM, ovalbumin (Ova), inactivated O1 Manisa FMDV vaccine antigen (FMDV, 0.001 µg ml^−1^), FMDV VP1 peptide (p252, 0.01 µg ml^−1^) or BHK-21 cell lysate (BHK-21, 0.001 µg ml^−1^). After 6 days, proliferation was assessed by [methyl-^3^H] thymidine incorporation. Results are expressed as the mean c.p.m. of triplicate determinations±sd. One representative dataset from five different animals is shown.

Carboxyfluorescein diacetate succinimydyl ester (CFDA SE) was used as a cell division marker for assessing the *in vitro* proliferation of PBMC isolated from O1 Manisa-vaccinated animals. By combining surface antigen and CFDA SE staining, we were able to determine the cell subsets involved in specific proliferation to both vaccine antigen and peptide 252 (p252). p252, within the VP1 region of FMDV, had previously been found to be a strong immunogenic peptide for PBMC harvested from MHC class II serotype A31 animals (Gerner *et al.*, 2007) and indeed Guzman *et al.* identified a MHC class I-restricted CD8^+^ T-cell epitope within this peptide (Guzman *et al.*, 2010). When analysing total PBMC (in the absence of specific surface antigen staining), there was a detectable increase in proliferative response after stimulation with FMDV vaccine antigen or FMDV p252 [Fig. 3 panel 1(a), 2(a) and 3(a)]. In the presence of medium alone there was very little non-specific proliferation of CD4^+^ and CD8^+^ T-cells [Fig. 3 panel 1(b) and 1(d)], however, a proportion of WC1^+^ γδ T-cells (3.5 %) did proliferate [Fig. 3 panel 1(c)]. Stimulation with FMDV antigen or specific peptide resulted in proliferation of CD4^+^, CD8^+^ and WC1^+^ γδ T-cells [Fig. 3 panels 2(b–d) and 3(b–d)]. Fig. 3 is an example of a dataset from one animal, similar profiles and magnitude of responses were observed for three animals. To confirm that the response was mediated primarily by CD4^+^ T-cells, the FMDV-specific proliferative responses of purified CD4^+^ T-cells were examined. Total PBMC showed a strong proliferative response to Pokeweed mitogen (PWM), ovalbumin (Ova), FMDV vaccine antigen (FMDV) and p252 but not to the negative control, BHK-21 cell lysate (Fig. 4a). With PBMC depleted of CD4^+^ T-cells, responses to Ova, FMDV vaccine antigen and p252 were no longer detectable and a much reduced response to the mitogen PWM was noticeable (Fig. 4b). Fig. 4(c) shows representative results obtained with purified CD4^+^ T-cells plus antigen presenting cells (APCs). These cells proliferated strongly, with c.p.m. values much higher than seen with total PBMC, in response to PWM, Ova, FMDV vaccine antigen and p252, and no response was elicited to the negative control. Consistent results have been shown with other animals (*n* = 5). These data indicate that CD4^+^ T-cells are the predominant component of the PBMC that respond specifically to FMDV antigen *in vitro*.

### Measurement of gamma interferon (IFN-γ) by ELISA and intracellular flow cytometry

**Fig. 5.**
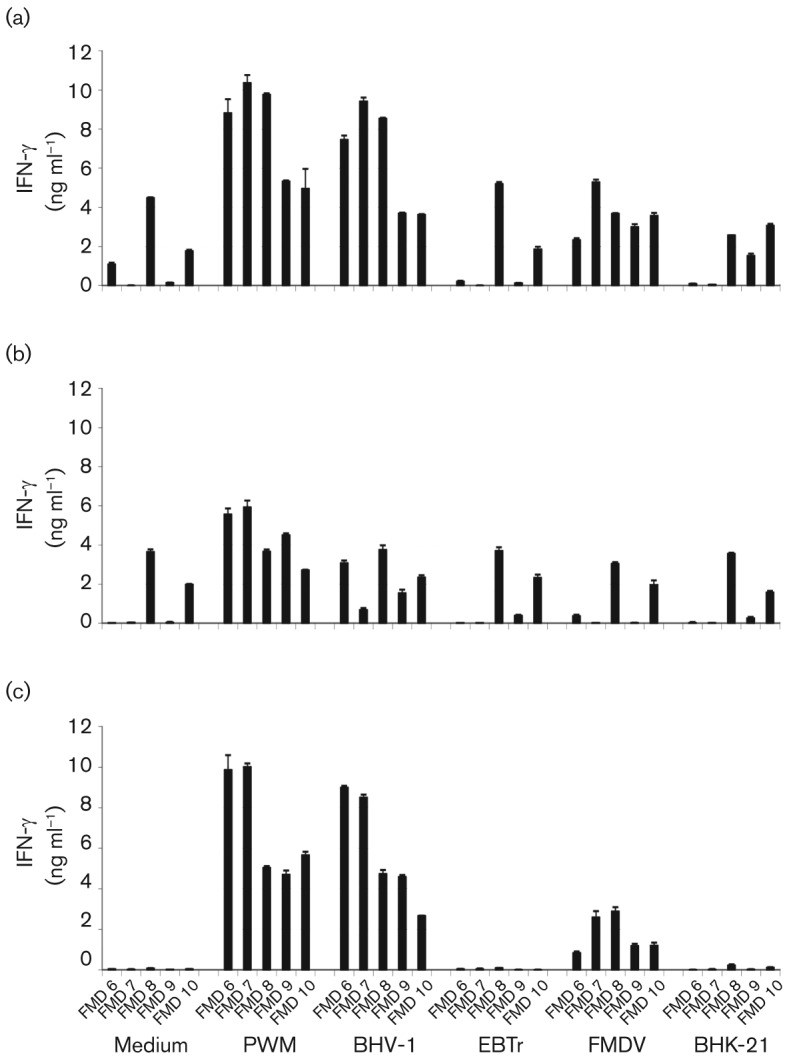
Quantification of IFN-γ production assessed by ELISA in response to *in vitro* restimulation with FMDV vaccine antigen. Five calves were vaccinated with commercial BHV-1 vaccine, and subsequently vaccinated with O1 Manisa FMD commercial vaccine (6 weeks later). Post-FMD vaccination, PBMC (a), PBMC depleted of CD4^+^ T-cells (b) and purified CD4^+^ T-cells+APCs (c) were cultured *in vitro* in the presence of medium alone or optimal concentrations of PWM, heat-inactivated BHV-1, EBTr cell lysate (EBTr), inactivated O1 Manisa FMDV vaccine antigen (FMDV, 0.1 µg ml^−1^) or BHK-21 cell lysate (BHK-21, 0.1 µg ml^−1^). After 5 days, supernatants were harvested and assayed for IFN-γ by sandwich ELISA. Results are expressed as the mean IFN-γ concentration (ng ml^−1^) of duplicate determinations±sd for each of the five vaccinated calves. For each culture condition, the five columns represent animals FMD 6–FMD 10.

We have shown by ELISA that IFN-γ was produced upon restimulation of both PBMC and purified CD4^+^ T-cells from FMD O1 Manisa-vaccinated cattle (FMD 1–FMD 10; Fig. 5 presents results from animals FMD 6–FMD 10). Non-specific IFN-γ production was also observed in supernatants from PBMC, from three out of the five animals examined, cultured in the presence of medium alone and relevant controls (Fig. 5a and b). This IFN-γ production is probably a result of the WC1^+^ γδ T-cells responding in an antigen-independent manner. However, analysis of purified CD4^+^ T-cells plus APCs stimulated with antigen demonstrated specific IFN-γ production with no background in the medium-alone stimulated supernatants (Fig. 5c).

**Fig. 6.**
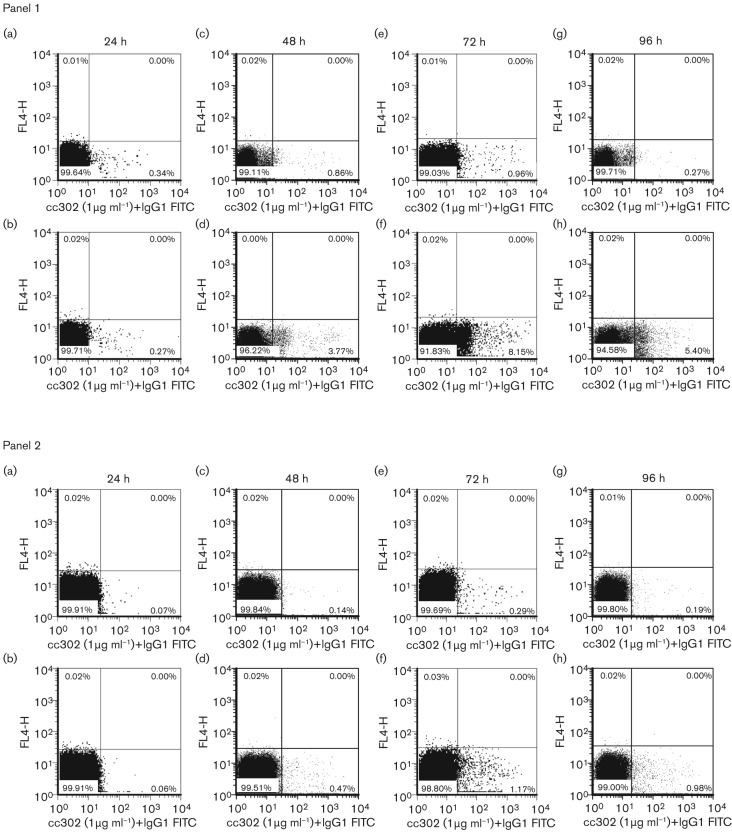
IFN-γ production assessed by intracellular flow cytometry in response to *in vitro* restimulation with FMDV vaccine antigen. Post-vaccination with O1 Manisa FMD commercial vaccine, PBMC (panel 1, 14 weeks post-vaccination) and purified CD4^+^ T-cells+APCs (panel 2, 24 weeks post-vaccination) were cultured *in vitro* for 24 (a and b), 48 (c and d), 72 (e and f) and 96 (g and h) h in the presence of medium+IL-12 (a, c, e and g) or optimal concentrations of inactivated O1 Manisa FMDV vaccine antigen (FMDV)+IL-12 (b, d, f and h). At the end of the culture period, the cells were permeabilized, stained intracellularly with either a mouse anti-bovine IFN-γ (cc302) or isotype-matched control mAb (TRT 1, data not shown) followed by incubation with FITC-conjugated goat anti-mouse IgG1 antibody (IgG1 FITC), and analysed by flow cytometry. The percentages of cells in each quadrant are illustrated. [Cells were only stained with FITC-labelled antibodies, the negative FL4-H fluorescence channel (*y*-axis) was only used for the purpose of creating the dot plots]. One representative dataset (animal FMD 7) from five (panel 1) and two (panel 2) different animals is shown.

In addition to the quantification in cell supernatants, intracellular flow cytometry was used to identify the specific cell subsets producing IFN-γ. IFN-γ could only be reliably detected by FACS after the addition of exogenous interleukin-12 (IL-12) into the culture system. When total PBMC were used in this assay we observed a population of cells producing a low level of IFN-γ, even in the presence of medium alone, making it difficult to formally determine whether these were responding cells, cells non-specifically producing IFN-γ or a result of non-specific staining [Fig. 6 panel 1(a), 1(c), 1(e) and 1(g)]. However, using purified CD4^+^ T-cells over the time-course of 96 h, little background was detected when cells were cultured in medium alone [Fig. 6 panel 2(a), 2(c), 2(e) and 2(g)] and increased quantities of IFN-γ-positive cells were detected in cells stimulated with FMDV vaccine antigen [Fig. 6 panel 2(b), 2(d), 2(f) and 2(h)]. These results clearly demonstrated that an effective functional T-cell response could be elicited by the vaccination.

## Discussion

To better characterize the immune response to FMD vaccines and to search for early markers predictive of induction of immune memory, we analysed the kinetics and magnitude of the antibody and cell-mediated immune responses to FMD vaccines.

Initially, we used a rigorous CD4^+^ T-cell depletion protocol in cattle to demonstrate a significant reduction in the VNTs post-vaccination in the absence of CD4^+^ T-cells. We have previously proposed that FMDV capsids are composed of T-dependent and T-independent B-cell epitopes. Specifically, we identified the G-H loop of VP1 as a T-dependent epitope, but have not characterized the specific nature of the T-independent epitopes. Results described here are consistent with our previous findings regarding the T-dependent antigenic nature of the G-H loop of FMDV capsids as G-H loop-specific antibodies were only detected in some CD4^+^ T-cell-depleted animals after the phase of CD4 depletion (Juleff *et al.*, 2009). In contrast to our CD4^+^ T-cell depletion study during FMDV infection, we showed that depletion of CD4^+^ T-cells during vaccination resulted in a significant reduction in FMDV-specific circulating antibody titres and a delay in detection of FMDV-specific IgG antibodies. Presumably, activation of CD4^+^ T-cells causes a cascade of cytokine production that supports enhancement of IgM and IgG production, vital signals missing during the transient depletion of CD4^+^ T-cells. We previously speculated that the activation of other cells by FMDV infection, for example WC1^+^ γδ T-cells, could result in the production of cytokines to support antibody production and class switching. In the present study, it would appear that vaccination with inactivated antigen does not activate these ‘bystander’ responses and antibody production is compromised by CD4^+^ T-cell depletion.

During the period of depletion, the already established T-cell response to bovine herpes virus-1 (BHV-1) was lost and failed to return upon the re-emergence of the CD4^+^ T-cells. Similar results have been reported with FMD-vaccinated animals by Naessens *et al.* (1998). The T-cell proliferative response in the CD4-depleted animals to PWM was also reduced throughout the period of depletion. Significantly, at the end of the period of T-cell depletion a specific T-cell proliferative response to FMDV could be detected, associated with an increase in VNTs in the depleted animals that became equivalent to the titres in the control group. Thus, these results confirm the requirement for a T-cell response to support maximum induction of neutralizing antibody titres. The induction of a specific T-cell response and increased antibody titres after re-emergence of circulating CD4^+^ T-cells suggest persistence of antigen in these animals during the depletion period. We have previously shown that FMDV persists on follicular dendritic cells after acute infection, which may also occur with inactivated antigen (Juleff *et al.*, 2008).

We then analysed both the antibody and cell-mediated responses in a prime–boost study with conventional FMD vaccine. A single dose of commercial vaccine was sufficient to expand CD4^+^ T-cells, whereby proliferative responses were readily detected in vaccinated animals as early as 7 days post-vaccination. These proliferative responses and VNTs could be readily boosted after a second dose of vaccine. Inactivated vaccines are known to induce protection of much shorter duration than natural infection, and require booster vaccination to maintain protective immunity (Pulendran & Ahmed, 2011). We examined whether the magnitude of the T-cell proliferative response could be correlated with the antibody response. One may predict that CD4^+^ T-cell responses and antibody responses should be closely associated because CD4^+^ T-cell help is required for the optimal activation and early clonal expansion of B-cells, and ultimately, for the generation of long-lived plasma and memory B-cells (Nutt & Tarlinton, 2011). However, due to a T-independent element of the FMDV antibody response, the poor correlation between VNTs and *in vitro* proliferative responses is not surprising. Antigen-specific cell proliferation by titrated thymidine incorporation or CFDA SE staining was a useful method to assess the functional capacity of cell populations using either whole PBMC or purified cell populations. Interestingly, consistent patterns of T-cell proliferation were observed when whole antigen or a specific MHC class II-restricted peptide were used in these studies.

By measuring cell proliferation by CFDA SE staining and flow cytometric analysis we identified the specific cell subsets responding to inactivated FMDV vaccine antigen. We could readily detect and quantify FMDV-specific CD4^+^ T-cell proliferation in PBMC and confirmed this result using purified CD4^+^ T-cells cultured with γ-irradiated PBMC as APCs. We also found that inactivated FMDV vaccine antigen stimulates proliferation of CD8^+^ T-cells and WC1^+^ γδ T-cells, similar to results described by Guzman *et al.* (2008) with CD8^+^ T-cells. It is likely that mechanisms of cross-presentation are contributing to the overall response, but this has not been investigated further. Importantly, the WC1^+^ γδ T-cell proliferation was abrogated when CD4^+^ T-cells were removed from PBMC prior to stimulation. We suggest WC1^+^ γδ T-cell proliferation *in vitro* is not an antigen-specific response but a consequence of CD4^+^ T-cell activation, which may explain why WC1^+^ γδ T-cells readily proliferate in peptide-stimulated PBMC cultures.

Further characterization of the antigen-specific CD4^+^ T-cell response was attempted by measuring IFN-γ production. Antigen-specific IFN-γ production by CD4^+^ T-cells could be measured in the cell culture supernatants of purified CD4^+^ T-cells cultured with irradiated APCs. However, in total PBMC cultures, non-specific IFN-γ production made it difficult to identify the magnitude of antigen-specific IFN-γ responses. In conclusion, it would be difficult to correlate IFN-γ production with antibody response or protection when using total PBMC populations. Instead, it may be possible to utilize IFN-γ production data using cell culture supernatants from purified CD4^+^ T-cells, although purification of cells from large groups of animals would be time consuming and expensive.

Subsequently, we investigated whether intracellular IFN-γ production, as assessed by flow cytometry, could be used to quantify CD4^+^ T-cell responses to FMDV antigen. During assay development, we found it necessary to add exogenous IL-12 to the culture medium to detect IFN-γ-producing cells. We have shown that all the major T-cell subsets, CD4^+^, CD8^+^ and WC1^+^ γδ^+^, produce IFN-γ by FACS (data not shown), which is consistent with our observations of IFN-γ production in cell culture supernatants, as detected by ELISA. However, although we can detect specific and quantifiable IFN-γ production in purified CD4^+^ T-cells we were unable to clearly quantify and observe these rare antigen-specific CD4^+^, IFN-γ^+^ T-cells in total PBMC populations by flow cytometry.

In summary, we have shown clearly that virus neutralizing antibody responses to inactivated FMDV vaccine antigen are compromised by ablation of CD4^+^ T-cell responses. We provide more support to our previous conclusion that T-dependent and T-independent B-cell epitopes are present on FMDV capsids. We also show that we can measure specific CD4^+^ T-cell proliferation in PBMC in response to FMDV antigen, but there is no significant correlation between the T-cell response and antibody titre. Increasing the duration of immunity of FMD vaccines is a major goal of significant practical importance and we conclude from our studies that enhancing the CD4^+^ T-dependent cellular response to FMDV capsids will potentially result in enhanced production of neutralizing antibodies.

## Methods

### 

#### *In vivo* depletion of CD4^+^ T-cells and FMD vaccination.

Two groups of three randomly assigned calves, approximately 30 days old, were used in this study. Animals were vaccinated against BHV-1 with Tracherine (Pfizer) and Bovilis IBR Marker Live vaccine (MSD Animal Health, Milton Keynes, UK), boost vaccinated 8 days later and proliferative responses measured prior to CD4^+^ T-cell depletion. Two weeks later, each animal received either an anti-CD4 (CD4^+^ T-cell depleted group: FMD 48, 51 and 52) or an isotype-matched control (non-depleted group: FMD 47, 49 and 50) mAb intravenously for a period of 4 days, starting the day before FMD vaccination (cc8 and TRT3 IgG2a-negative control, respectively) (Serotec). Total dose of antibody over the 4 day period was 2.5 mg kg^−1^ of body weight. All animals were vaccinated with A22 Iraq FMD commercial vaccine (Merial Animal Health Ltd, Harlow, UK) (two antigen payloads of 4 ml per animal) when percentages of circulating CD4^+^ T-cells were below background in the depleted group (determined by flow cytometry). PBMC were obtained at various time points prior to and following the intravenous injection of calves with cc8 or TRT3 (IgG2a) mAbs and stained with an anti-CD4 mAb (cc30, IgG1) or its isotype-matched negative control TRT1 mAb, followed by incubation with FITC-conjugated goat anti-mouse IgG1 antibody (1/200; SouthernBiotec). Viable cells (100 000) per sample were analysed using a FACScan flow cytometer (BD Biosciences). Conventional PBMC lymphoproliferation assays were performed weekly and antibody titres were measured daily according to the methods outlined below.

#### Animal vaccination.

A series of longitudinal studies of the T-cell response to FMD vaccine were undertaken at the Institute for Animal Health, Compton. Cattle were 3- to 6-month old conventionally reared Holstein-Friesians, housed in secure accommodation. A control immunization regime was followed, whereby animals FMD 1–FMD 5 were immunized with Ova (Sigma) in incomplete Freund’s adjuvant and animals FMD 6–FMD 10 were vaccinated against BHV-1 with Bovilis IBR Marker Live vaccine. In each instance, between 4 and 6 weeks later conventional monovalent O1 Manisa FMD oil adjuvant vaccine (Merial Animal Health Ltd) was given intramuscularly, according to the manufacturer’s instructions. Eight out of ten calves were boost-vaccinated either 6 or 11 weeks later as detailed in Table 2.

#### Measurement of antibody responses, PBMC and CD4^+^ T-cell purification and cell proliferation using thymidine incorporation.

See online Supplementary material.

#### Measurement of T-cell subset proliferation by CFDA SE labelling and staining.

The antigen-specific T-cell response was also assessed in specific T-cell subsets. PBMC with the different antigens and controls were set up in a similar manner as described above for the conventional lymphoproliferation assays, except the PBMC were labelled with Vybrant carboxyfluorescein diacetate succinimidyl ester (CFDA SE) cell tracer (Invitrogen) immediately after their isolation. Briefly, cells were diluted to 1×10^7^ ml^−1^ in pre-warmed PBS and CFDA SE added to give a final concentration of 5 µM. Manufacturer’s instructions were followed and labelled cells were adjusted to the appropriate concentration for use in the assay. Twelve replicate wells per stimulation were set up and incubated as described above. Six days later, the cells were harvested from culture wells, replicate wells per stimulation pooled, fixed and stained using appropriate cell surface markers coupled to Allophycocyanin (APC) [anti-CD4: cc8 mAb, anti-WC1^+^ γδ: cc15 mAb, anti-CD8: cc63 mAb] to identify the lymphocyte subpopulations, for further analysis by two-colour flow cytometry.

#### Measurement of IFN-γ by ELISA and intracellular flow cytometry.

For the determination of IFN-γ levels in cell supernatants by ELISA, cells were cultured as described above for 5 days. Supernatants were harvested and stored at −70 °C before assaying for IFN-γ according to the methods of Hope *et al.* (2002). Results were expressed as the mean IFN-γ concentration (ng ml^−1^) of duplicate determinations±sd. For intracellular IFN-γ detection, cells were cultured as described above for 24, 48, 72 or 96 h with medium alone or inactivated FMDV vaccine antigen in the presence of 1 U IL-12 ml^−1^. The protein transport inhibitor Brefeldin A (final concentration 10 µg ml^−1^; Merck) was added for the final 20 h of the incubation period to prevent secretion and thus allow accumulation of the cytokine inside the cell. At the end of the culture period, replicate wells for each stimulation were pooled, fixed, permeabilized and stained with 1 µg ml^−1^ mouse anti-bovine IFN-γ mAb (clone cc302; Serotec), followed by incubation with FITC-conjugated goat anti-mouse IgG1 antibody (1/200; SouthernBiotec) and acquired using a FACScan flow cytometer (BD Biosciences). Analysis was performed using the FCS Express software (*de novo* software). Viable cells (50 000) per sample were acquired.

#### Statistical methods.

Potential correlation between c.p.m. and VNT was explored by computing Spearman partial correlations for these variables, controlling for the effects of day, timing of booster and antigen (FMD 1–FMD 10) or day and CD4-depletion status (FMD 47–FMD 52). Changes in VNT, IgG1, IgG2, IgM and G-H loop peptide antibody levels (*y*) assessed by ELISA over time (*t*) were described using a Gompertz function,

y(t)=κexp⁡{−exp⁡[−β(t−β)]}

where β is the rate of increase, δ is the time at which the maximum rate of increase occurs and κ is the maximum level. Parameters for each animal were estimated by fitting the above curve to the log-transformed data using the method of least-squares. Estimates for the individual animals were then compared using Kruskal–Wallis tests to identify significant (*P*<0.05) differences amongst CD4-depleted and non-depleted animals. Differences in FMDV-specific VNT, IgG1, IgG2, IgM and G-H loop peptide antibody levels between CD4-depleted and non-depleted cattle were further explored by comparing levels at individual time points using Kruskal–Wallis tests.

#### Ethics of experimentation.

All experiments were carried out in accordance with the Home Office Guidance on the Operation of the Animals (Scientific Procedures) Act 1986 and associated guidelines, and approved by the local Ethics Committee at the Institute for Animal Health. No adverse reaction or toxicities attributable to either BHV-1 or FMD vaccination procedures were observed.
